# Rhinovirus 3C Protease Facilitates Specific Nucleoporin Cleavage and Mislocalisation of Nuclear Proteins in Infected Host Cells

**DOI:** 10.1371/journal.pone.0071316

**Published:** 2013-08-07

**Authors:** Erin J. Walker, Parisa Younessi, Alex J. Fulcher, Robert McCuaig, Belinda J. Thomas, Philip G. Bardin, David A. Jans, Reena Ghildyal

**Affiliations:** 1 Centre for Research in Therapeutic Solutions, University of Canberra, Canberra, Australian Capital Territory, Australia; 2 Department of Biochemistry and Molecular Biology, Monash University, Clayton, Victoria, Australia; 3 Monash Institute of Medical Research and Monash Lung & Sleep, Monash Medical Centre, Clayton, Victoria, Australia; Stanford University, United States of America

## Abstract

Human Rhinovirus (HRV) infection results in shut down of essential cellular processes, in part through disruption of nucleocytoplasmic transport by cleavage of the nucleoporin proteins (Nups) that make up the host cell nuclear pore. Although the HRV genome encodes two proteases (2A and 3C) able to cleave host proteins such as Nup62, little is known regarding the specific contribution of each. Here we use transfected as well as HRV-infected cells to establish for the first time that 3C protease is most likely the mediator of cleavage of Nup153 during HRV infection, while Nup62 and Nup98 are likely to be targets of HRV2A protease. HRV16 3C protease was also able to elicit changes in the appearance and distribution of the nuclear speckle protein SC35 in transfected cells, implicating it as a key mediator of the mislocalisation of SC35 in HRV16-infected cells. In addition, 3C protease activity led to the redistribution of the nucleolin protein out of the nucleolus, but did not affect nuclear localisation of hnRNP proteins, implying that complete disruption of nucleocytoplasmic transport leading to relocalisation of hnRNP proteins from the nucleus to the cytoplasm in HRV-infected cells almost certainly requires 2A in addition to 3C protease. Thus, a specific role for HRV 3C protease in cleavage and mislocalisation of host cell nuclear proteins, in concert with 2A, is implicated for the first time in HRV pathogenesis.

## Introduction

Human Rhinoviruses (HRVs) belong to the picornavirus family of small positive strand RNA viruses. HRVs are the main viral causative agents of upper respiratory tract infections and also cause the majority of asthma exacerbations [1], [2], making understanding of the molecular mechanisms underlying HRV pathogenesis of great medical importance. HRV infection is initiated by binding to host cell plasma membrane receptors (usually, intercellular adhesion molecule-1 or the low density lipoprotein receptor) followed by conformational changes in the HRV capsid leading to release of the viral genome into the cytoplasm of the infected cell [3]. The viral genome is then translated into a polyprotein, which is subsequently processed via intra- and intermolecular cleavages into various structural and non-structural proteins, mediated by the HRV-encoded proteases, 2A and 3C [3]. As in the case of poliovirus, HRV infection results in the shut-down of key host cell processes [3], where recruitment of several host proteins by the picornavirus internal ribosome entry site (IRES) element results in their non-availability for cellular functions, enabling optimal viral polyprotein translation [3], [4], [5], [6].

Many viruses disrupt host cell nucleocytoplasmic trafficking or appropriate it for their own use [7], [8], [9], [10], [11], [12] probably as a strategy to limit anti-viral responses that require efficient nucleocytoplasmic trafficking [13], [14]. Disruption of host cell nuclear transport would have profound effects on the intracellular signalling required for normal cell function [15], thus contributing to the observed pathogenesis [16], [17], [18]. Recent work has shown that HRV infection results in the cleavage of translation and transcription factors, as well as the nucleoporin (Nup) proteins that make up the nuclear pore, the only avenue for transport into and out of the nucleus [19], [20], [21]; cleavage of Nups such as Nup153 and Nup62, can result in inhibited nuclear import [20], [21], [22] leading to the mislocalization of host cell proteins. HRV 2A protease was previously implicated in the cleavage of Nup62 [22] in HRV-infected cells, but the protease responsible for cleavage of the other Nups that are degraded in HRV infection, and the molecular changes that result in the mislocalisation of nuclear proteins in HRV infected cells have not yet been elucidated.

Here we use transfected and HRV-infected cells to demonstrate that 3C protease is responsible for cleavage of Nup153 and nucleolin in infected cells. We also demonstrate that HRV16 infection leads to cleavage of the phenylalanine-glycine (“FG”) repeat–containing Nups 153, 98 and 62, but not of non-FG Nups such as Nups 133 or 93, which constitute the structural core of the nuclear pore. Finally, we show that ectopic expression of active but not inactive HRV16 3C protease results in mislocalisation of the nuclear splicing factor SC35 and nucleolin. Our results establish for the first time that HRV 3C protease has a specific role in subverting transport through the nuclear pore in HRV16 infection.

## Methods

### Antibodies

The primary antibodies for the following proteins were used for Western analysis and immunofluorescence (IF): anti-Nup62 (BD Biosciences #610497, used at dilutions of 1∶2000 for Western and 1∶1000 for IF), anti-Nup93 (Santa Cruz #292099, used at 1∶1000), anti-Nup98 (Abcam #45584, used at 1∶1000 for Western and Santa Cruz sc74578 used at 1∶500 for IF), anti-Nup133 (Santa Cruz #376699, used at 1∶1000), anti-Nup153 (Abcam #96462, used at 1∶1000 for Western and 1∶500 for IF), anti-hnRNP-A1 (Santa Cruz #56700, used at 1∶2000 for Western and 1∶500 for IF), anti-hnRNP-C1/C2 (Santa Cruz #32308, used at 1∶2000 for Western and 1∶500 for IF), anti-nucleolin (Abcam #22758, used at 1∶3000 for Western and 1∶1000 for IF), anti-SC35 (Sigma #4045, used at 1∶500 for IF), and anti-α/β-tubulin (Cell Signalling Technology #2148, used at 1∶1000 for Western). Antibodies to 3C protease and VP2 were kindly provided by S. Amineva (Madison, WI) [19] and W. Lee (Madison, WI) [23] respectively.

### Cell Culture and Infection

Ohio-HeLa cells (provided by Bo Lin, Biota Holdings) and COS-7 cells (CRL-1651, American Type Culture Collection) were grown in high glucose DMEM supplemented with 10% heat inactivated Foetal Bovine Serum (FBS) and antibiotics (penicillin, streptomycin, neomycin) at 37°C in a humidified atmosphere of 5% CO_2_. Rhinovirus serotype 16 (HRV16), used for all infection experiments, was a gift from E. Dick and W. Busse (Madison, WI). Viral stocks were prepared by infecting subconfluent monolayers of Ohio-HeLa cells at a multiplicity of infection (MOI) of 1 by absorption for 1 h with occasional rocking, followed by replacement of the medium with fresh DMEM supplemented with 2% FBS and antibiotics. Once extensive cytopathic effects were observed, infected cultures were frozen at −80°C to release virus [24]. Cultures were thawed, vortexed and clarified of cellular debris by centrifugation for 15 min at 3,500 rpm. Infectious virus was titrated on Ohio-HeLa cells by standard TCID50 protocol and titre calculated using the Spearman-Karber equation [25].

### Plasmid Constructs and Transfection

The coding sequence for HRV16 3C was amplified by PCR from the full length HRV16 genome [26] and recombined into pDONR207 to generate a Gateway compatible entry clone which was then recombined into the Gateway compatible episomally replicating vector, pEPI-DESTC, to encode GFP-3C for mammalian cell expression [10], [27]. An inactive form of HRV16 3C (termed 3Cinac) was generated, where the active site cysteine (Cys 147) [3] was mutated to alanine by site-directed mutagenesis and recombined into pEPI-DESTC as for wild type, to encode GFP-3Cinac. Plasmid DNA was transfected into subconfluent monolayers of COS-7 cells using either Lipofectamine 2000 (Invitrogen) as recommended by the manufacturer, or TransIT-2020 (Mirus), where half the suggested volume of TransIT-2020 reagent was used. Transfected cells were cultured for 18 h before being processed for Western analysis or IF.

### *In vitro* Protease Cleavage Assay

Confluent monolayers of Ohio-Hela cells were lysed at 10^7^ cells/ml in cold RIPA buffer (50 mM Tris, pH 8.0, 150 mM sodium chloride, 1.0% NP-40, 0.5% sodium deoxycholate, 0.1% sodium dodecyl sulphate) for 30 min on ice with rocking, followed by centrifugation at 13,300 rpm for 1 min to remove cell debris. Lysate equivalent to 1.5×10^6^ cells was incubated with 4U of recombinant HRV 3C protease (Novagen) at 30°C for various times prior to heating at 100°C for 5 min in Laemmli buffer to stop the reaction [28], followed by Western analysis.

### Western Analysis

Overnight subconfluent cultures of Ohio-HeLa cells with or without infection with HRV16 at an MOI of 1 were lysed at different times by incubation in RIPA buffer containing protease and phosphatase inhibitors (Roche) for 30 min on ice as described above, prior to heating at 100°C for 5 min in Laemmli buffer. COS-7 cells transfected to express GFP-3C or GFP-3Cinac were enriched by sorting for GFP fluorescence using a FACS Aria II (Becton Dickinson) Cell Sorter using BD FACS Diva v6.1.4 and lysed in cold RIPA buffer (containing protease and phosphatase inhibitors) followed by denaturation in Laemmli buffer as above.

Cell lysates (see above) were subjected to SDS-polyacrylamide electrophoresis using pre-cast gradient (4–20%) or 10% acrylamide gels (Bio-Rad, TGX gels) followed by Western transfer to nitrocellulose membranes in Tris-Glycine-ethanol buffer (25 mM Tris-HCl, 192 mM Glycine, 20% Ethanol) for 90 min at 400 mA. Blots were stained with Ponceau S (Sigma) to confirm transfer and then blocked for 1 h in 4% skim milk (Diploma) in PBS (10 mM Na_2_HPO_4_, 1.7 mM KH_2_PO_4_, pH 7.2, 2.7 mM KCl, 137 mM NaCl), prior to incubation with different primary antibodies diluted in 1% skim milk in PBS-T (PBS containing 0.1% Tween 20) overnight at 4°C with rocking. After washing in PBS-T, blots were incubated with specific secondary antibodies conjugated to horseradish peroxidase diluted 1∶5000 in 1% skim milk in PBS-T, followed by washing and detection of bound antibodies with Enhanced Chemiluminiscence (ECL, Perkin Elmer) and exposure to X-ray film (Kodak film). Films were scanned (Epson 2450 Photo Scanner) and digital images analysed using ImageJ to estimate relative protein levels. Where required, blots were stripped using stripping buffer (2% SDS, 62.5 mM Tris-HCl pH 6.8, 114.4 mM β-mercaptoethanol) at 50°C for 10 min, followed by washing in PBS-T, blocking in 4% skim milk in PBS and reprobing using different primary antibodies as required.

### Immunofluorescence (IF)

Overnight subconfluent monolayers of Ohio-HeLa cells grown on glass coverslips (Proscitech, #1) were infected with HRV16 at an MOI of 1 or left uninfected (mock) and fixed with 4% formaldehyde in PBS followed by permeabilisation of cell membranes with 0.2% Triton X-100 in PBS at various times p.i. [27]; an MOI of 1 was used to enable direct comparison of both infected and uninfected cells in the same microscopic field (see Results section). COS-7 cells transfected to express either GFP-3C or GFP-3Cinac were fixed similarly 18 h after transfection. Cells were incubated with primary antibodies diluted in PBS for 30 min, washed twice in PBS, incubated with species specific secondary antibodies conjugated to Alexa Fluor 488 or Alexa Fluor 568 diluted 1∶1000 in PBS for 30 min, and then washed twice in PBS and mounted using ProLong Gold mounting media with DAPI (Invitrogen). Digitised fluorescent cell images were collected using a Nikon Ti Eclipse confocal laser-scanning microscope (CLSM) with Nikon 60x/1.40 oil immersion lens (Plan Apo VC OFN25 DIC N2; optical section of 0.5 µm) and the NIS Elements AR software.

## Results

### HRV16 Infection Leads to Cleavage of Nuclear Pore Components and Other Nuclear Proteins

**Figure 1:**
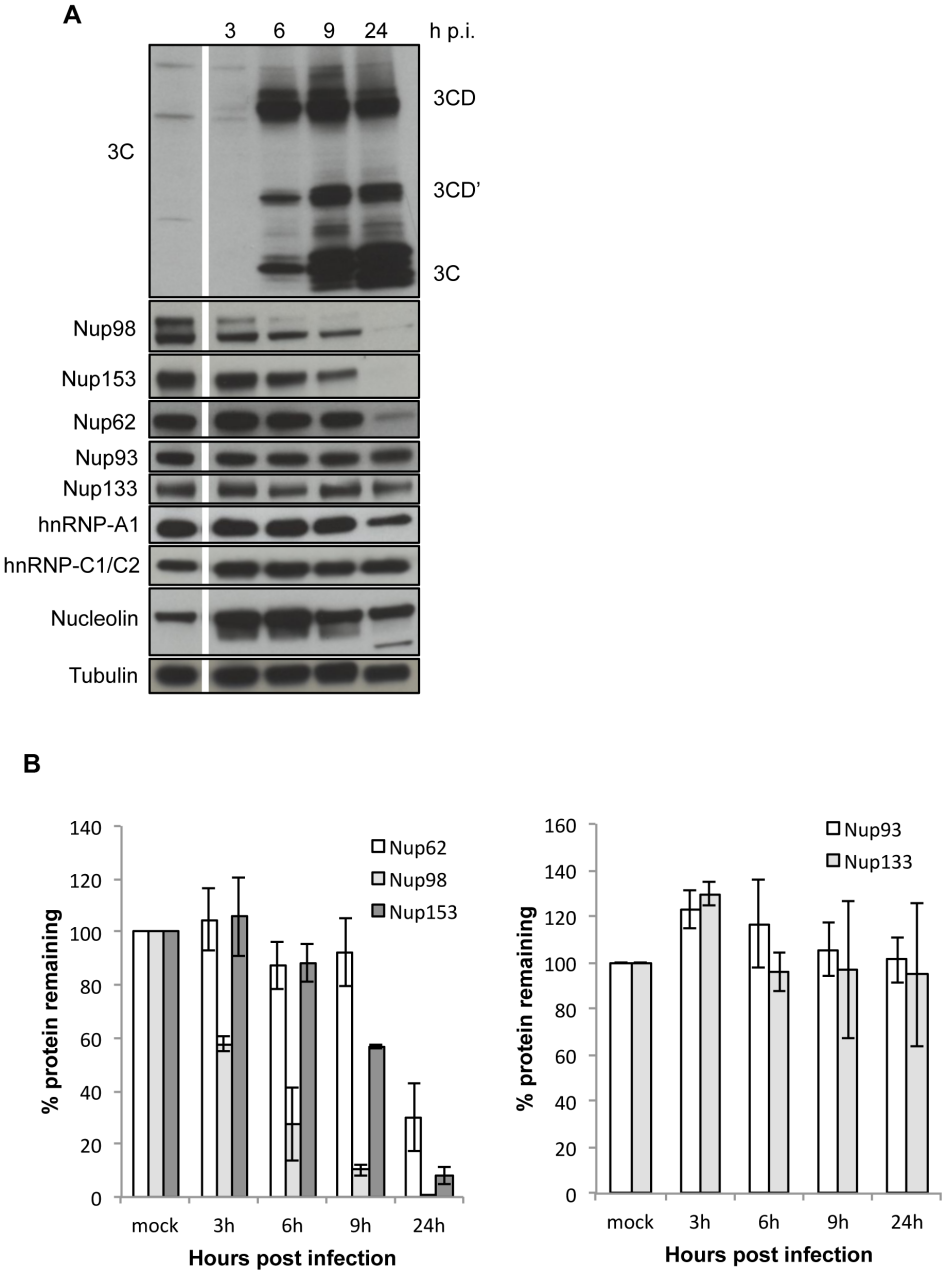
Specific nucleoporins are degraded in HRV16-infected cells. (**A**) Ohio-HeLa cells were infected without (mock) or with HRV16 (MOI of 1) and cells lysed using RIPA buffer containing protease and phosphatase inhibitors at the time points shown. Cell lysates were subjected to SDS-PAGE on 4–20% gradient gels and Western analysis using the indicated primary antibodies/horseradish peroxidise-conjugated secondary antibodies and enhanced chemiluminescence (Perkin Elmer). The specificity of the antibodies is indicated on the left. Bands corresponding to 3C, 3CD’ and 3CD are indicated on the right. p.i. - post-infection. (**B**) Results for densitometric analysis of FG-Nup protein bands (left) and non-FG-Nups (right) such as those shown in (A), where data were normalised to the corresponding values for tubulin, relative to the corresponding values for the mock sample. Densitometric analyses were performed using Image J; values represent the mean (± SD) from two independent experiments.

The effect of HRV16 on nuclear proteins/nuclear pore components was initially assessed by Western analysis over time in HRV16-infected cells, followed by quantitation by densitometry (Figure 1). Cell lysates were analysed for 3C protease, Nup62, Nup98, Nup153, Nup93, Nup133, heterogeneous nuclear ribonucleoprotein particle (hnRNP) A1 and C1/C2 proteins and nucleolin, with tubulin as a loading control. 3C protease and its precursor 3CD were clearly evident in cell lysates from 6 h p.i., as was 3CD’, the product of a 2A cleavage within 3CD [19]. In contrast to the non-FG Nups 93 and 133 which showed negligible changes in levels, cleavage of Nup153 was detectable even at 6 h p.i., coinciding with the appearance of 3C protease in the cells, and progressively increased up to 24 h p.i., at which time, Nup153 was undetectable by Western. That loss of Nup153 was due to enzymatic cleavage was supported by the appearance of a cleavage product at 65 kDa (Figure S1A). Nup62 cleavage appeared to occur more slowly, being first detected at 6–9 h p.i., and with substantial reduction only evident at 24 h p.i.; a cleavage product was observed at 25 kDa from 6 h p.i. (Figure S1B). Cleavage of Nup98 was evident at 3 h p.i., preceding the detection of 3C and continued progressively until 24 h p.i., at which time very little intact Nup98 remained. Multiple bands were detected for Nup98, consistent with previously published results [29], with no distinct cleavage products evident (Figure S1C). The above observations were confirmed by quantitative analysis, results showing that Nup98 was the first FG Nup to be degraded (with only about 10% of the protein remaining at 9 h p.i.), followed by Nup153 (with about 90% cleavage evident at 24 h p.i.), and then, Nup62, with about 30% of Nup62 still remaining at 24 h p.i. (Figure 1B, left panel). In contrast, the levels of the non-FG Nups 93 and 133, as well as hnRNP-A1, hnRNP-C1/C2 and nucleolin, like tubulin, did not show any marked indication of cleavage (Figure 1A) in HRV16 infected cells, although an exception to this was an additional band of c. 80 kD observed for nucleolin at 24 h p.i., suggestive of some cleavage at later times p.i. Quantitative analysis (Figure 1B, right panel) also confirmed the lack of cleavage of non-FG Nups in HRV16 infection.

**Figure 2:**
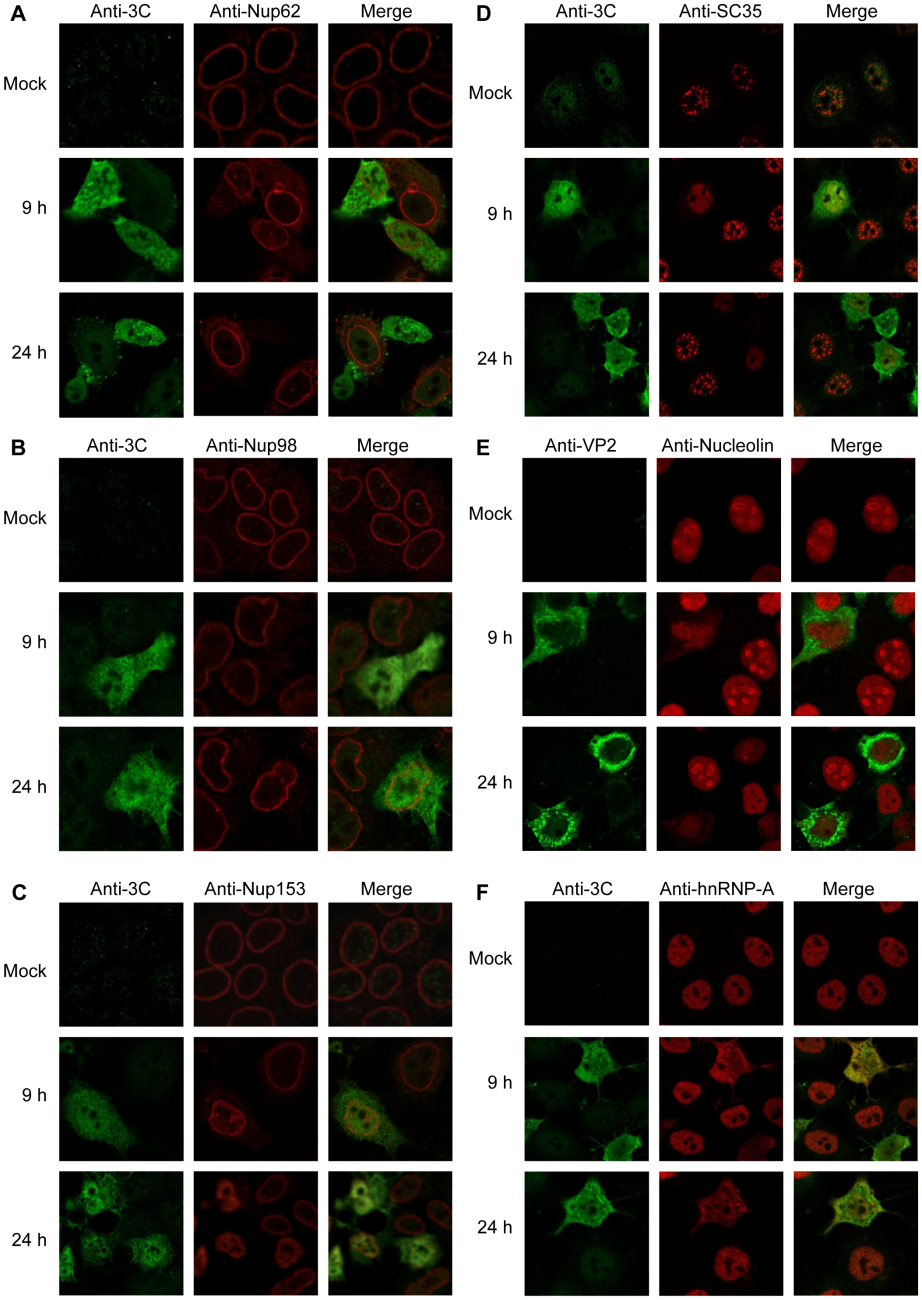
HRV16 infection leads to mislocalisation of nuclear proteins. Ohio-HeLa cells grown on coverslips were infected without (mock) or with HRV16 as per Figure 1; cells were fixed at the indicated times and permeabilized, and then probed with the indicated pairs of primary antibodies, followed by Alexa 488 and Alexa-568 conjugated secondary antibodies. Fluorescence was imaged by CLSM (see Materials and Methods). In each panel, images on the left depict localisation of HRV16 proteins (green channel) and the images in the middle depict localisation of cellular proteins (red channel), with the merged image on the right.

In parallel, indirect IF was performed, where overnight monolayers of Ohio-HeLa cells grown on coverslips were infected without (mock) or with HRV16, fixed at the various time points and probed for 3C (Figure 2ABCDF) or VP2 (Figure 2E) in combination with Nup62 (Figure 2A), Nup98 (Figure 2B), Nup153 (Figure 2C), SC35 (Figure 2D), nucleolin (Figure 2E) or hnRNP-A1 (Figure 2F). Consistent with the Western analysis, indirect IF showed that infection with HRV16 resulted in progressively reduced levels of fluorescence specific for Nup62, with reduced staining in infected compared to uninfected cells at 9 h p.i., and almost complete absence of staining in a number of cells at 24 h p.i. (Figure 2A). In contrast, and inconsistent with the results obtained by Western analysis, staining for Nup98 revealed no marked change over the course of infection (Figure 2B), which could be a consequence of the antibody epitope remaining preserved and *in situ* despite cleavage (see [30]). Staining for Nup153 revealed mislocalisation of this protein in infected cells beginning at 9 h p.i. and continuing to 24 h p.i., whereby Nup153 was no longer found discretely at the nuclear membrane, but distributed throughout the nucleus (Figure 2C). In the case of SC35, and in contrast to infection with HRV14 [21], the typical “nuclear speckle” pattern of staining became diffuse in HRV16 infected cells and at 24 h p.i., SC35 staining was almost absent (Figure 2D). HRV16 infection also resulted in the normally strongly nucleolar staining of nucleolin changing to become diffusely nuclear in infected cells from 9 h. p.i., and weak at 24 h p.i. (Figure 2E). Finally, consistent with previous observations for HRV14 infected cells [20], hnRNP-A1 was found to alter localisation from strongly nuclear, to diffuse localisation throughout the cell in HRV16 infected cells (Figure 2F); a similar result was observed for hnRNP-C1/C2 (data not shown). Our analysis shows that concomitant with cleavage of FG- but not the structural non-FG Nups, HRV16 infection results in the mislocalisation of various nuclear and nucleolar components.

### Active HRV16 3C is Sufficient to Effect Cleavage of FG-Nups and Mislocalisation of Nuclear Proteins in Transfected Cells

**Figure 3:**
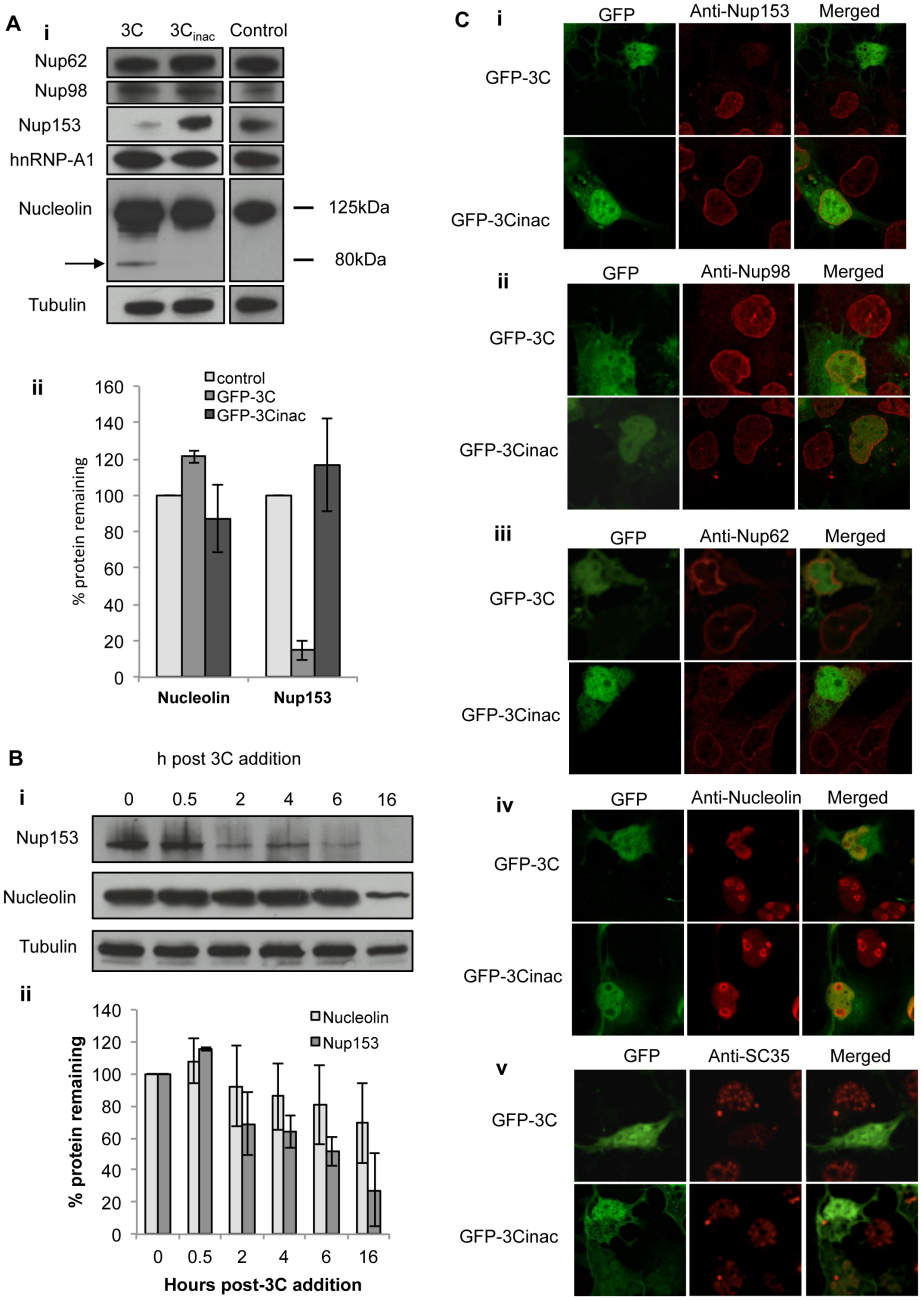
Active 3C protease is sufficient to degrade nuclear proteins/nucleoporins in transfected cells. (**Ai**) COS-7 cells transfected to express either GFP-3C or GFP-3Cinac were trypsinized 18 h after transfection, harvested in ice-cold PBS and FACS sorted to collect GFP-expressing cells. Cells were then lysed using RIPA buffer plus protease and phosphatase inhibitors and lysates were subjected to Western analysis as per Figure 1A; untransfected cells were lysed similarly and used as control. The antibodies used are shown on the left of the figure. The arrow in the nucleolin blot denotes a clear cleavage product, with the approximate molecular weight (kDa) indicated on the right. (**Aii**) Results for densitometric analysis of nucleolin and Nup153 protein bands such as those shown in Figure 3Ai, where data were normalised to the corresponding value of tubulin, relative to the corresponding value for the control sample. Densitometric analyses were performed using ImageJ; values represent the mean (+ SD) from two independent experiments (**Bi**) Ohio-Hela whole cell lysates were incubated with 4 units of HRV14 3C protease at 37°C for the indicated incubation times, subsequent to SDS-PAGE on 10% gels and Western analysis as per Figure 1A. (**Bii**) Plot of densitometric analysis of protein bands in (Bi), where data were normalised to the corresponding values for tubulin, relative to 0 h samples. Densitometric analyses were performed using Image J and values were the mean of two different experiments (± SD). (**C**) COS-7 cells transfected to express either GFP-3C or GFP-3Cinac were fixed and permeabilized 18 h post-transfection, and immunostained as described in Figure 2 with the indicated primary and Alexa-568 conjugated secondary antibodies. Fluorescence was imaged by CLSM (see Materials and Methods). In each panel, images on the left depict localisation of HRV16 proteins (green channel) and the images in the middle depict localisation of cellular proteins (red channel), with the merged image on the right.

To assess the extent to which the above changes in HRV 16 infected cells could be solely attributed to the activity of 3C, we transfected COS-7 cells to express GFP fused HRV16 3C or 3Cinac, a mutant derivative where the active site cysteine (cys-147) [3] has been mutated to alanine to result in a lack of protease activity, and then GFP positive cells FACS sorted, lysed and their levels of Nups/nuclear proteins assessed by Western analysis. Equivalent numbers of lysed, non-transfected COS-7 cells were included as an untreated control. We observed cleavage of Nup153, as well as nucleolin (with a clear proteolytic cleavage product evident at c. 80 kD indicated by the arrow in Figure 3Ai), in cells expressing GFP-3C, but not in those expressing GFP-3Cinac or untransfected cells; tubulin levels were not different among the three cell groups, implying the specificity of the results. Quantitative analysis (Figure 3Aii) confirmed the results, indicating markedly reduced Nup153 in cells expressing GFP-3C compared to control cells or cells expressing inactive 3C. In contrast to results obtained in HRV16 infection, we observed no evidence for cleavage of either Nup62 or Nup98 (Figure 3Ai) in cells expressing GFP-3C or GFP-3Cinac.

To determine whether the observed cleavage of Nup153 and nucleolin could be a direct result of 3C activity or a consequence of 3C protease-activation, we examined the effect of purified HRV14 3C protease on whole cell lysates. HRV14 and 16 3C proteases are believed to possess the same substrate specificity based on similarity between HRV16- (shown here) and HRV14- infected cells [21] in terms of Nup degradation/relocalisation of nuclear proteins, and the comparable lack of 3C activity specifically against Nup62 observed here for HRV16 3C, and by others for HRV14 3C protease [31]. Our analysis revealed cleavage of Nup153 within 2 hours of 3C addition, and near complete cleavage by 16 h (Figure 3Bi), whereas nucleolin was intact up until 16 h. Quantitative analysis (Figure 3Bii) confirmed these results, with a c. 30% reduction in Nup153 levels evident at 2 h after 3C addition, and over 70% at 16h. Together, the results suggest that active 3C is sufficient to cleave Nup153, and to induce cleavage of nucleolin in live cells, but is not sufficient to cleave Nups62 or 98.

The results were extended by performing IF for Nup62, Nup98, Nup153, nucleolin, and SC35 on cells transfected to express either GFP-3C or -3Cinac (Figure 3C). Significantly, reduced nuclear envelope staining was observed for Nup153 in cells expressing GFP-3C, compared to cells expressing GFP-3Cinac (Figure 3C, i), the clear implication being that active 3C is sufficient to cleave Nup153 at the nuclear pore in living cells. No change in expression or localisation of Nup62 or Nup98 was observed in cells expressing GFP-3C compared to cells expressing GFP-3Cinac, consistent with the Western analysis indicating a lack of effect of 3C (Figure 3C, ii, iii). Nucleolin and SC35 showed altered localisation in the presence of GFP-3C compared to GFP-3Cinac; specifically, nucleolin became more prominent in the nucleus and was absent from the nucleoli (Figure 3C, iv) while SC35 changed from its normal nuclear speckle pattern to a more diffuse pattern in GFP-3C expressing cells (Figure 3C, v). No change in the localisation of any hnRNP proteins was observed by IF after transfection with GFP-3C (Figure S2). Overall, our analysis indicates that 3C’s effects on the nuclear pore lead to the mislocalisation of some but by no means all nuclear proteins.

## Discussion

This is the first study to clearly establish that HRV16 3C protease is the likely mediator of Nup153 cleavage, and mislocalisation of SC35 and nucleolin in HRV16 infected cells. In particular, the results from using different approaches indicate that proteolytically active 3C protease is sufficient to effect cleavage of Nup153. This activity appears to be complementary to that of HRV16 2A protease in cleaving Nup62 cleavage [22], which contributes to hnRNP relocalisation.

Our study is the first to assess the effect of HRV16 infection on FG- and non-FG-Nups. Interestingly, HRV16 infection resulted in cleavage of all FG-Nups but none of the non-FG-Nups analysed. This finding is significant in the context of the functions attributed to these two classes of Nups; FG-Nups are involved directly in the movement of cargo through the nuclear pore complex (NPC) while the non-FG Nups have a structural role [32]. Our finding provides the underlying mechanism for the observations in previous reports that the overall structure of the NPC remains intact in picornavirus infection even while nuclear transport is disrupted through a so-called “leaky” pore [18], [33].

Significantly, expression of active GFP-tagged 3C in transfected cells resulted in the mislocalisation of the splicing factor SC35 from nuclear speckles to become diffusely nucleoplasmic (see Figure 3C), precisely paralleling SC35 localisation in HRV16-infected cells (see Figure 2D). This has not been observed in previous studies using HRV14 or poliovirus [17], [21], but may reflect a serotype-specific difference with respect to HRV16. Since SC35 plays a key role in elongation of nascent mRNA transcripts [34], determination of RNA splice junctions and spliceosome assembly [35], its mislocalization in HRV infected cells is likely to contribute significantly to host cell shut-down.

The hnRNPs A1 and C1/C2 shuttle in a signal dependent fashion between the nucleus and cytoplasm, and are believed to be involved in mRNA splicing and export and mRNA splicing and stability, respectively [36], with both playing a role in translational control of mRNAs that contain IRESs [37], such as those found on HRV genomes. HnRNP-A1 in particular is reported to bind to the IRES of HRV2 [37], with its nuclear import pathway dependent on transportin [38], [39] known to be disrupted in HRV14 and poliovirus infection [17], [21], while hnRNP-C1/C2 is a non-shuttling protein previously shown to mislocalise to the cytoplasm in HRV/poliovirus infection [17], [21]. We observed a similar mis-localisation of hnRNP-A1 and hnRNP-C1/C2 to the cytoplasm in HRV16 infected cells. That we observed no change in hnRNP protein localisation in cells transfected to express 3C protease alone implies that 2A is required to effect its mislocalisation.

The nucleolar RNA binding shuttling protein nucleolin appears to be a common target of virus action, having previously been shown to mislocalise to the cytoplasm upon infection with HRV14 [21], poliovirus [29], [40], feline calicivirus [41], and even herpes simplex virus 1 (HSV-1); here we confirm similar results for HRV16 infection, and demonstrate mislocalisation from the nucleolus to the nucleoplasm in cells expressing active GFP-3C protease (see Figure 3C), concomitant with cleavage of the protein, either directly by 3C or in a 3C protease-dependent manner (see Figure 3). These results require more detailed investigation, but imply that active 3C may directly modulate nucleolin localisation, potentially through cleavage of nucleolin itself, as well as effects on Nups/the nuclear pore.

**Figure 4:**
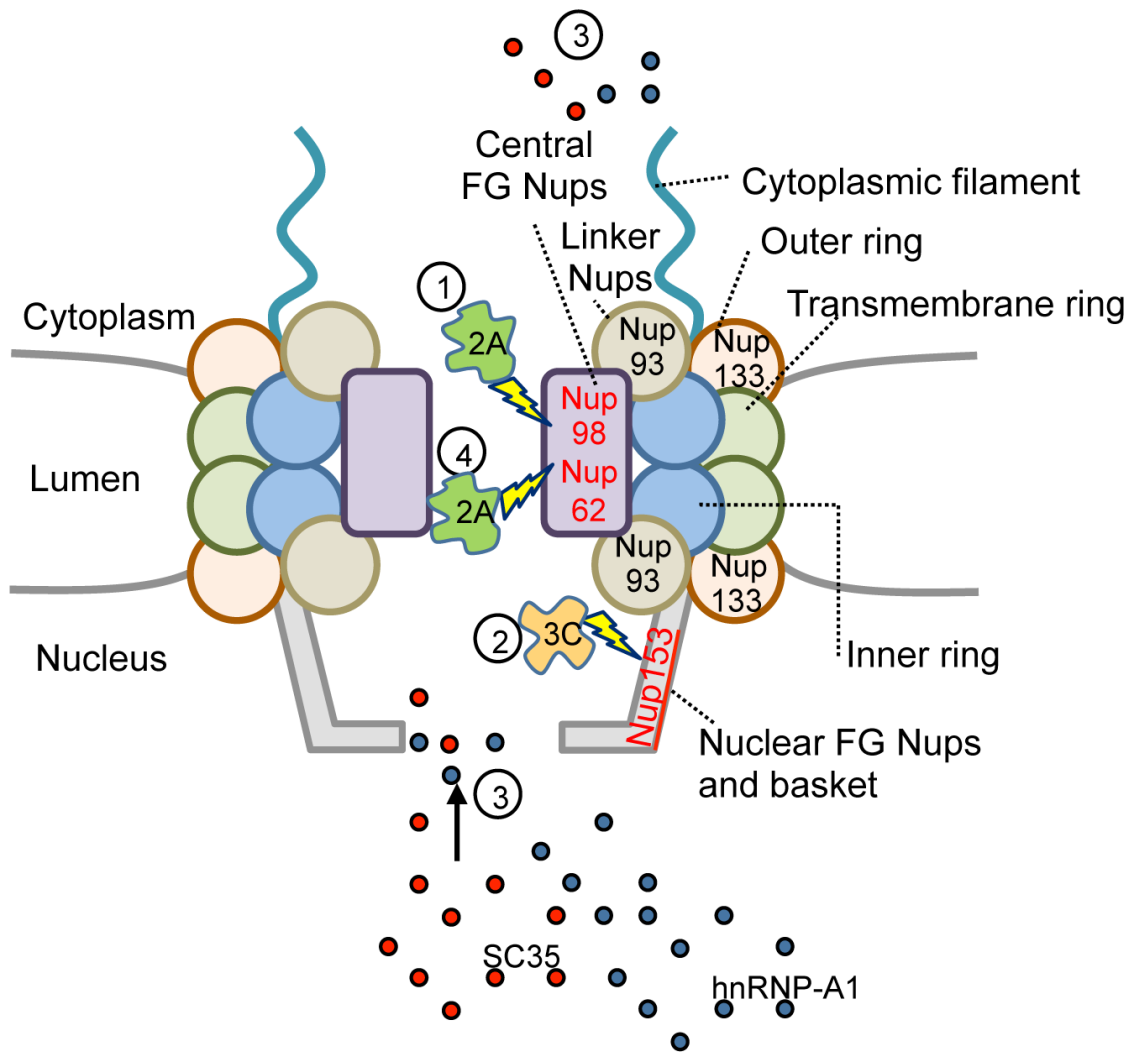
Schematic representation of proposed FG-Nup cleavage by 2A and 3C proteases during HRV16 infection. During infection with HRV16, the initial cleavage event within the nuclear pore is mediated by 2A protease and leads to Nup98 cleavage (1). Cleavage of Nup153 by 3C protease occurs at approximately 6–9 h p.i. correlating with the localisation of 3C in the nucleus (2). Mislocalisation of SC35 from nuclear speckles to the nucleus and cytoplasm and mislocalisation of hnRNP-A1 from the nucleus to the cytoplasm is detected at 6 h p.i. (3). Cleavage of Nup62 by 2A protease occurs later, between 9–24 h p.i. (4). Nups that were cleaved during infection are shown in red type while Nups that were unaffected during infection are shown in black type.

Taken together with previous reports from our and other groups, our data lead us to propose a model of HRV16 infection (Figure 4) whereby 2A protease mediates the initial cleavage of Nup98 early during infection (starting at 3 h p.i.), with cleavage of Nup153 by 3C protease following at about 6–9 h p.i., at which time 3C is localised to the nucleus. At this point it appears that the nuclear pore is sufficiently degraded to allow mislocalisation of hnRNP-A1 from the nucleus to the cytoplasm, and that 3C protease activity within the nucleus significantly affects localisation of SC35 within nuclear speckles and nucleolar localisation of nucleolin. At 9–24 h p.i. Nup62 is cleaved by 2A protease and is likely a late event during infection due to Nup62 being present in a complex with other Nups and not being easily accessible. Clearly, detailed examination of Nup cleavage during infection, outside the scope of the present study, is required to establish the precise timing of the different events, and how they relate to one another. That the results shown here for HRV16-induced Nup degradation have relevance to other HRV serotypes is strongly implied by the studies of Gustin and Sarnow [21] showing similar Nup degradation by HRV14. Additionally, given the high sequence conservation of 3C protease across serotypes [42], the substrate specificity should be comparable across serotypes, as has been shown for 2A protease [43].

In conclusion, we show for the first time that 3C protease mediates cleavage of Nup153 and effects the mislocalisation of SC35 and nucleolin observed in HRV16 infection. The cleavage of other Nups and mis-localisation of hnRNP-A1 probably require the added protease activity of 2A. The study is also the first to show that structural non-FG Nups are not cleaved during HRV16 infection. Future work in this laboratory is focussed on elucidating the specific sequence of events in the infected cell mediated by these proteases, and how they synergise successfully in effecting host cell shutoff.

## Supporting Information

Figure S1 **Nup cleavage products observed during HRV16 infection.** Over-exposure of the Western blots presented in Figure 1A reveals accumulation of specific cleavage products for (A) Nup153, (B) Nup62, but no obvious cleavage products for (C) Nup98 The arrows in (A) and (B) show the location of cleavage products. Full length and cleavage products are indicated on the right and molecular weights (kDa) are indicated on the left. The ‘*’ indicates additional bands that are often observed for these antibodies.
(TIF)

Figure S2 **3C protease activity alone does not lead to mislocalisation of hnRNP proteins.** COS-7 cells transfected to express either GFP-3C or GFP-3Cinac were fixed and permeabilized 18 h post-transfection, and immunostained with the indicated primary and Alexa-568 conjugated secondary antibodies. Fluorescence was imaged by CLSM (see Materials and Methods). In each panel, images on the left depict localisation of HRV16 proteins (green channel) and the images in the middle depict localisation of cellular proteins (red channel), with the merged image on the right.
(TIF)
