# Supplementary figures and images for: Rhinovirus 3C Protease Facilitates Specific Nucleoporin Cleavage and Mislocalisation of Nuclear Proteins in Infected Host Cells
Source: PLoS One. 2013 Aug 7;8(8):e71316. doi: 10.1371/journal.pone.0071316 (PMC3737158; doi:10.1371/journal.pone.0071316)

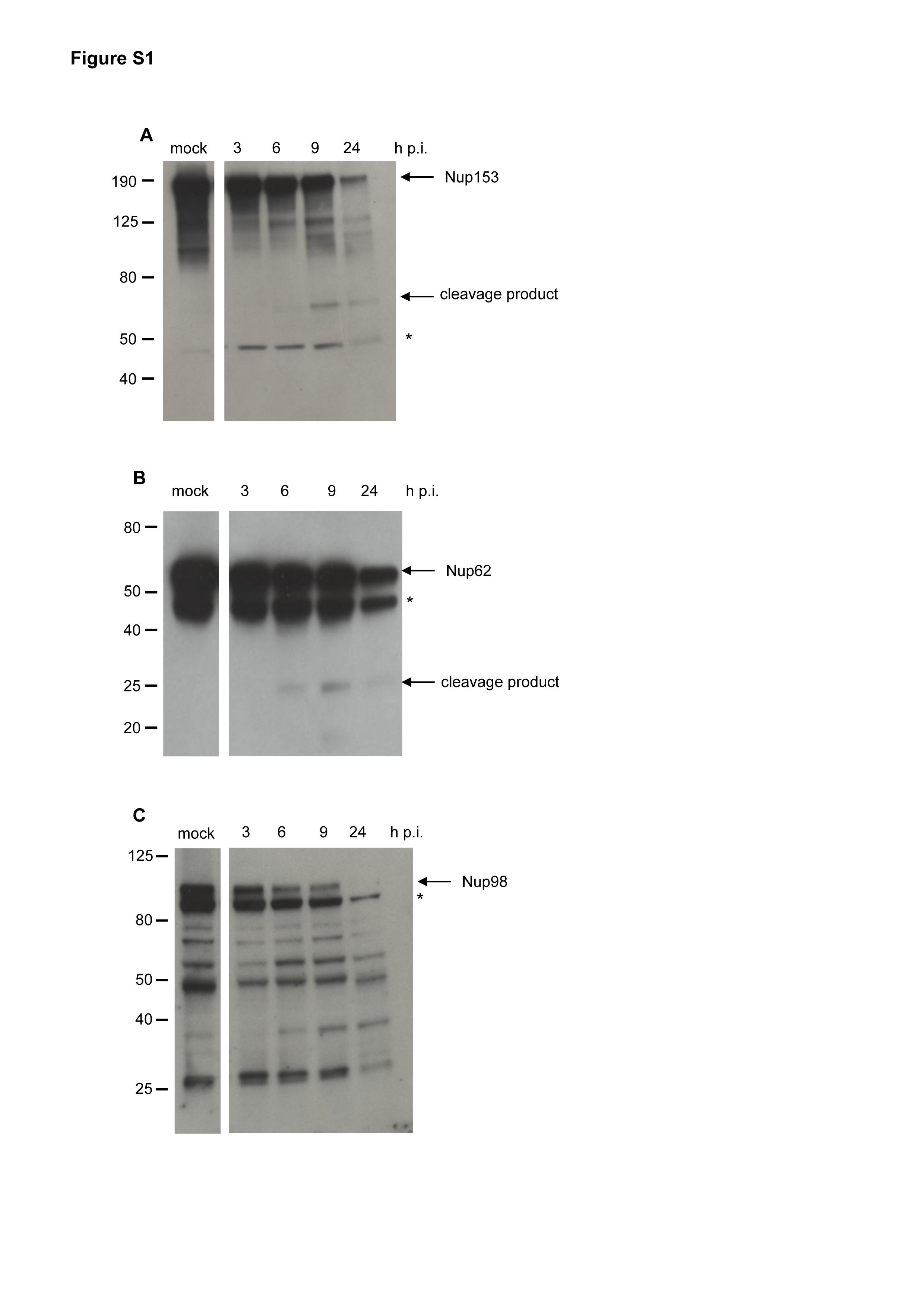

Supplement: Figure S1 — Nup cleavage products observed during HRV16 infection. Over-exposure of the Western blots presented in Figure 1A reveals accumulation of specific cleavage products for (A) Nup153, (B) Nup62, but no obvious cleavage products for (C) Nup98 The arrows in (A) and (B) show the location of cleavage products. Full length and cleavage products are indicated on the right and molecular weights (kDa) are indicated on the left. The ‘*’ indicates additional bands that are often observed for these antibodies. (TIF) [file pone.0071316.s001.tif]

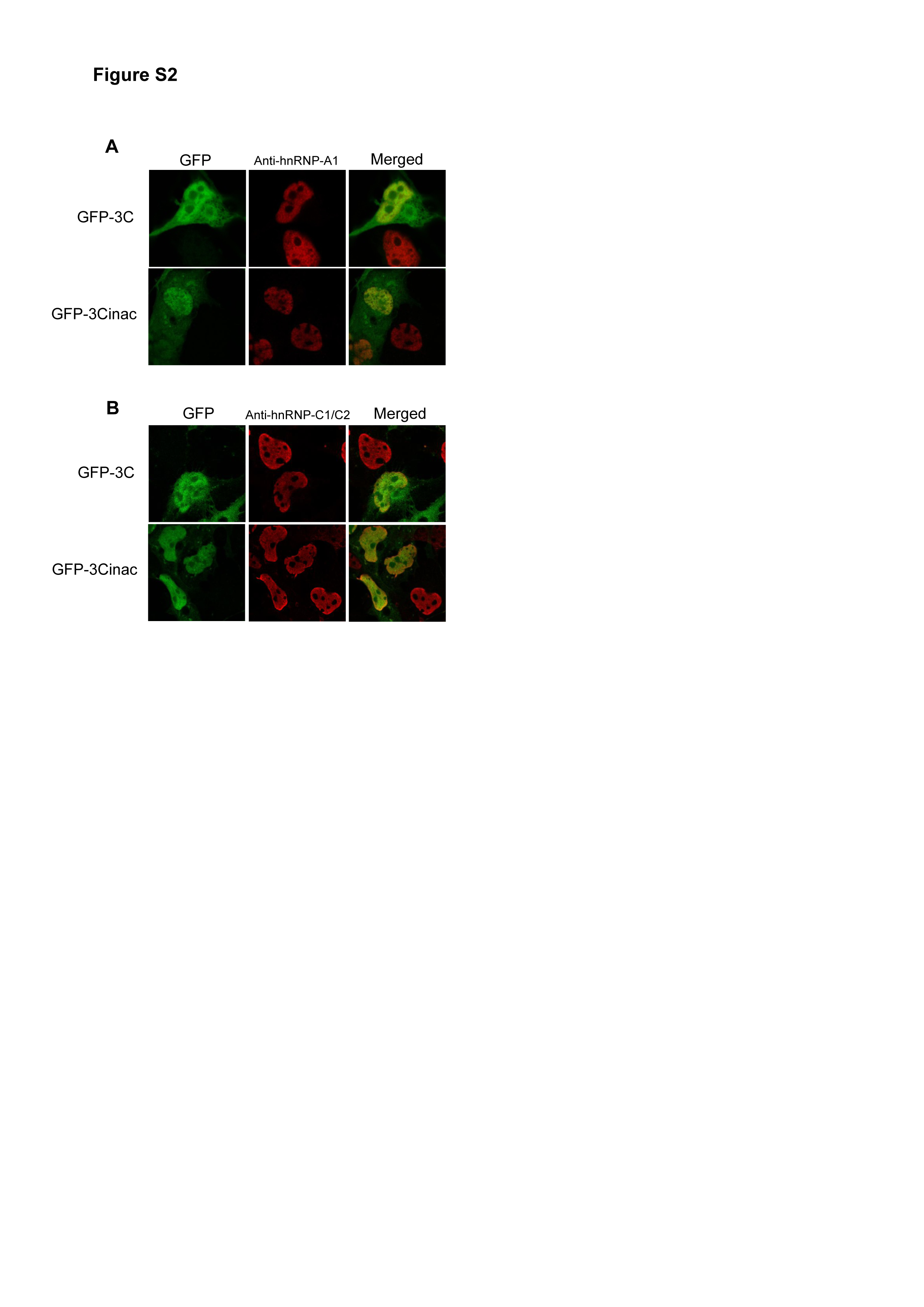

Supplement: Figure S2 — 3C protease activity alone does not lead to mislocalisation of hnRNP proteins. COS-7 cells transfected to express either GFP-3C or GFP-3Cinac were fixed and permeabilized 18 h post-transfection, and immunostained with the indicated primary and Alexa-568 conjugated secondary antibodies. Fluorescence was imaged by CLSM (see Materials and Methods). In each panel, images on the left depict localisation of HRV16 proteins (green channel) and the images in the middle depict localisation of cellular proteins (red channel), with the merged image on the right. (TIF) [file pone.0071316.s002.tif]
